# Supplementary material for: Mapping the evolution of fertility support policies in China: A content and instrumental analysis
Source: PLoS One. 2025 Oct 9;20(10):e0332137. doi: 10.1371/journal.pone.0332137 (PMC12510515; doi:10.1371/journal.pone.0332137)
Supplement: S1 Appendix — (ZIP) [file pone.0332137.s001.zip › S1 Appendix. 226 original policy documents/37-国家卫生健康委办公厅关于印发托育机构负责人培训大纲(试行)和托育机构保育人员培训大纲(试行)的通知(FBM-CLI-4-5055415).doc]

国家卫生健康委办公厅关于印发托育机构负责人培训大纲(试行)和托育机构保育人员培训大纲(试行)的通知

**发布部门：** [国家卫生健康委员会](https://www.pkulaw.com/law/chl?Aggs.IssueDepartment=60321) [机构沿革](javascript:void(0))

**发文字号：**国卫办人口函〔2021〕449号

**发布日期：**2021.08.19

**实施日期：**2021.08.19

**时效性：** [现行有效](https://www.pkulaw.com/law/chl?Aggs.TimelinessDic=01)

**效力级别：** [部门工作文件](https://www.pkulaw.com/law/chl?Aggs.EffectivenessDic=XE0303)

**法规类别：** [卫生综合规定](https://www.pkulaw.com/law/chl?Aggs.Category=09601)

国家卫生健康委办公厅关于印发托育机构负责人培训大纲（试行）和托育机构保育人员培训大纲（试行）的通知
（国卫办人口函〔2021〕449号）

各省、自治区、直辖市及新疆生产建设兵团卫生健康委：
　　为深入贯彻《[国务院办公厅关于促进3岁以下婴幼儿照护服务发展的指导意见](https://www.pkulaw.com/chl/434b24b8b5769383bdfb.html)》（国办发〔2019〕15号）精神，切实加强托育服务人才队伍建设，我委组织制定了《托育机构负责人培训大纲（试行）》和《托育机构保育人员培训大纲（试行）》。现予以印发，请遵照执行。
　　一、强化统筹规划
　　各级卫生健康部门要统筹做好托育机构负责人和保育人员岗位培训总体规划，确立托育机构负责人和保育人员岗位培训制度，将其作为急需紧缺人员纳入培训规划，分批次开展培训工作。

　　二、建设培训资源
　　各级卫生健康部门要遴选一批基础较好的优质教材和课程资源，推进托育机构负责人和保育人员培训相关教材建设，充分发挥高校、行业学（协）会和示范托育机构力量，开发高质量培训指导教材和资源库。

　　三、加强培训监管
　　各级卫生健康部门要对托育机构负责人和保育人员培训机构加强监管，建立定期评估机制，形成动态管理、有进有出的竞争管理机制。
　　附件：1.托育机构负责人培训大纲（试行）
　　2.托育机构保育人员培训大纲（试行）

国家卫生健康委办公厅
　　2021年8月19日

　　附件1

　　托育机构负责人培训大纲（试行）

　　一、培训对象
　　拟从事或正在从事托育机构管理工作的负责人。

　　二、培训方式
　　采用理论和实践相结合、线上与线下相结合的方式。培训总时间不少于60学时，其中理论培训不少于40学时，实践培训不少于20学时。

　　三、培训目标
　　通过培训，使参训托育机构负责人端正办托思想，正确理解贯彻党和国家的托育服务方针政策；规范办托行为，具备履行岗位职责必备的基本知识与能力；增强管理能力，能够科学组织与管理托育机构。
　　（一）端正办托思想
　　1.熟悉并执行托育服务相关政策法规，增强法治意识，履行岗位职责，遵守行业规范。
　　2.具备良好的职业道德，树立正确科学的儿童观、保育观。
　　（二）规范办托行为
　　1.理解托育机构管理岗位要求，能够建立信息管理、健康管理、疾病防控和安全防护监控制度，制定安全防护、传染病防控等应急预案，确保婴幼儿的安全和健康。
　　2.根据婴幼儿身心发展特点和规律，制订科学的保育方案，合理安排一日生活和活动，提供支持性环境，满足婴幼儿健康成长的需要。
　　（三）提升管理能力
　　1.规划托育机构发展，加强保育的组织与管理，增强对保育人员的指导、检查和评估，引领托育机构质量提升。
　　2.与家庭、社区密切合作，整合各方资源支持托育机构保育工作，向家长、社区提供照护服务和指导服务，帮助家庭增强科学育儿能力。

　　四、培训内容
　　（一）理论培训内容
　　1.法律法规和政策文件。《[中华人民共和国未成年人保护法](https://www.pkulaw.com/chl/883cecd7fe38636dbdfb.html)》《[中华人民共和国母婴保健法](https://www.pkulaw.com/chl/34423ea29b054d35bdfb.html)》《[中华人民共和国母婴保健法实施办法](https://www.pkulaw.com/chl/a91f6202b8996971bdfb.html)》《[中华人民共和国食品安全法](https://www.pkulaw.com/chl/ac73cbe3ed780d7fbdfb.html)》《[托儿所幼儿园卫生保健管理办法](https://www.pkulaw.com/chl/c2e282d5a2ded4d9bdfb.html)》等相关法律法规，《[国务院办公厅关于促进3岁以下婴幼儿照护服务发展的指导意见](https://www.pkulaw.com/chl/434b24b8b5769383bdfb.html)》《托育机构设置标准（试行）》《托育机构管理规范（试行）》《托儿所、幼儿园建筑设计规范》《[建筑设计防火规范](https://www.pkulaw.com/chl/43869fc3bf1af0c5bdfb.html)》《托育机构登记和备案办法（试行）》《托育机构保育指导大纲（试行）》《托育机构婴幼儿伤害预防指南（试行）》《婴幼儿喂养健康教育核心信息》等相关政策文件。
　　2.职业道德。职业认同，岗位职责，行业规范，儿童权利，婴幼儿家庭合法权益，心理健康知识。
　　3.专业理念。儿童观，保育观，与家庭、社区合作共育观念，医育结合理念。
　　4.规范发展。登记备案，托育服务协议签订，收托健康检查，收托信息管理，信息公示，机构发展规划，机构发展反思与改进。
　　5.卫生保健知识。室内外环境卫生，设施设备、用品、材料等卫生消毒，婴幼儿常见疾病、传染病、伤害的预防与控制，科学喂养与膳食添加，睡眠环境与照护，晨午检与全日健康观察，体格锻炼，心理行为保健，工作人员健康管理。
　　6.安全防护。安全消防知识，食品安全知识，场地设施，婴幼儿适龄的家具、用具、玩具、图书、游戏材料配备要求，安全防护措施和检查，突发事件应急预案与处理。
　　7.保育管理。婴幼儿生理、心理发展知识，一日生活和活动安排与组织，生活与卫生习惯培养，动作、语言、认知、情感与社会性等方面保育要点，户外活动要求与组织，游戏安排与组织，环境创设与利用。
　　8.人员队伍管理。人员配备与资格要求，人员劳动合同签订，人员合法权益保障，人员职位晋升与工作激励，人员岗前培训与定期培训，人员安全与法治教育，人员专业发展规划，人员心理健康管理。
　　9.外部关系。家长会议、家长接待与咨询、家长委员会、家长开放日等与家庭合作相关的要求与策略，向家庭、社区提供照护服务和指导服务的内容与策略，配合主管部门业务指导的内容与要求。
　　（二）实践培训内容
　　1.机构规范设置。托育机构场地、建筑设计、室内外环境、设施设备、图书与游戏材料等规范设置的实践观摩与学习。
　　2.日常管理制度。信息管理、健康管理、膳食管理、疾病防控、安全防护、人员管理、人员培训、财务管理、家长与社区联系等制度的建立与实施，年度工作计划制定与定期报告，托育机构质量评估制度的建立与落实。
　　3.保育活动组织。入托、晨检、饮食、饮水、如厕、盥洗、睡眠、游戏、离托等一日生活安排与指导，动作、语言、认知、情感与社会性等保育活动组织与指导，环境创设，照护服务日常记录和反馈，保育人员工作的检查和评估。
　　4.应急管理训练。婴幼儿常见伤害急救基本技能，防范、避险、逃生、自救的基本方法，消防、安全保卫等演练，突发意外伤害的处理程序，安全突发事件应急处理程序。

　　五、培训原则
　　（一）岗位胜任原则
　　培训应以托育机构负责人岗位要求为重点，通过系统培训引导与自主学习反思相结合的方式，促进托育机构负责人明晰岗位工作任务，具备胜任岗位职责的基本知识与能力。
　　（二）需求导向原则
　　培训应以托育机构负责人在管理工作中的重点与难点为出发点，综合考虑岗位需求和发展需要，按需施教，优化培训内容，确保托育机构负责人学以致用、用以促学、学用相长。
　　（三）多元方式原则
　　培训可通过专题讲座、网络研修、研讨交流、案例分析、返岗实践等多元方式，借助互联网等手段，推动托育机构负责人理论学习和现场观摩相结合、线上学习与线下研修相结合，提高培训的便捷性、有效性。

　　六、培训考核
　　培训考核内容分为理论考试和实践技能考核两部分，各级卫生健康部门负责对培训效果进行抽查。
　　附件2

　　托育机构保育人员培训大纲（试行）

　　一、培训对象
　　拟从事或正在从事托育机构保育工作的保育人员。

　　二、培训方式及时间
　　采用理论和实践相结合、线上与线下相结合的方式。培训总时间不少于120学时，其中理论培训不少于60学时，实践培训不少于60学时。

　　三、培训目标
　　通过培训，使参训保育人员熟悉托育服务法规与政策，树立法治意识与规范保育思想；学习保育工作的基本技能与方法，强化安全保育意识；掌握婴幼儿早期发展与回应性照护的知识与策略，提升科学保育素养。
　　（一）增强规范保育意识
　　1.熟悉托育服务相关政策法规，遵守保育人员岗位职责和基本规范；
　　2.具备良好的职业道德和专业认同感；树立正确的保育观念，坚持儿童优先，保障儿童权利。
　　（二）掌握安全保育方法
　　1.切实做好安全防护工作，最大限度地保护婴幼儿的安全和健康；
　　2.掌握婴幼儿卫生保健、生活照料等保育工作的基本方法和操作规范。
　　（三）提升科学保育能力
　　1.合理安排婴幼儿的生活和活动，具备促进婴幼儿早期发展的能力，满足婴幼儿身体发育和心理发展的需要；
　　2.掌握与家庭及社区沟通合作的技巧，提供科学育儿指导，及时进行专业反思。

　　四、培训内容
　　（一）理论培训内容
　　1.法律法规和政策文件。《[中华人民共和国未成年人保护法](https://www.pkulaw.com/chl/883cecd7fe38636dbdfb.html)》《[中华人民共和国母婴保健法](https://www.pkulaw.com/chl/34423ea29b054d35bdfb.html)》《[中华人民共和国母婴保健法实施办法](https://www.pkulaw.com/chl/a91f6202b8996971bdfb.html)》《[托儿所幼儿园卫生保健管理办法](https://www.pkulaw.com/chl/c2e282d5a2ded4d9bdfb.html)》等相关法律法规；《[国务院办公厅关于促进3岁以下婴幼儿照护服务发展的指导意见](https://www.pkulaw.com/chl/434b24b8b5769383bdfb.html)》《托育机构设置标准（试行）》《托育机构管理规范（试行）》《托育机构保育指导大纲（试行）》《托育机构婴幼儿伤害预防指南（试行）》《婴幼儿喂养健康教育核心信息》等相关政策文件。
　　2.职业道德。职业规范，职业责任，儿童权利保护，专业认同，人文素养，心理健康等。
　　3.专业理念。儿童观，保育观，医育结合理念等。
　　4.卫生保健知识。卫生与消毒，物品管理，生长发育监测，体格锻炼，心理行为保健，婴幼儿常见病预防与管理，传染病预防与控制，健康信息收集。
　　5.安全防护。食品安全知识，环境与设施设备防护安全，婴幼儿常见伤害预防与急救，意外事故报告原则与流程等。
　　6.生活照料。各月龄营养与喂养要点，进餐照护，饮水照护，睡眠照护，生活卫生习惯培养，出行照护等。
　　7.早期发展支持。婴幼儿生理、心理发展知识，婴幼儿个体差异与支持，特殊需要婴幼儿识别与指导，活动设计与组织等。
　　8.沟通与反思。日常记录与反馈，与家庭、社区沟通合作，家庭、社区科学养育指导，保育实践反思等。
　　（二）实践培训内容
　　1.卫生消毒。活动室、卧室等室内外环境卫生清扫、检查和预防性消毒，抹布、拖布等卫生洁具的清洗与存放，床上用品、玩具、图书、餐桌、水杯、餐巾等日常物品的清洁与预防性消毒。
　　2.健康管理。晨午检及全日健康观察，运动和体格锻炼，健康行为养成，计划免疫宣传与组织等。
　　3.疾病防控。发热、呕吐、腹泻、惊厥、上呼吸道感染等常见疾病的识别、预防与护理，手足口、疱疹性咽炎、水痘、流感等婴幼儿常见传染病的识别、报告与隔离，贫血、营养不良、肥胖等营养性疾病，先心病、哮喘、癫痫等疾病婴幼儿的登记和保育护理。
　　4.安全防护。窒息、跌倒伤、烧烫伤、溺水、中毒、异物伤害、动物致伤、道路交通伤害等常见伤害急救技能，地震等重大自然灾害的逃生流程与演练，火灾、踩踏、暴力袭击等突发事件的预防与应急处理。
　　5.饮食照护。膳食搭配，辅食添加，喂养方法，进餐环境创设，进餐看护与问题识别，独立进餐、专注进食、不挑食等饮食习惯培养，辅助婴幼儿水杯饮水等。
　　6.睡眠照护。睡眠环境创设，困倦信号识别，睡眠全过程观察、记录与照护；规律就寝、独立入睡等睡眠习惯培养，睡眠问题的识别与应对，婴幼儿睡眠的个别化照护等。
　　7.清洁照护。刷牙、洗手、洗脸、漱口和擦鼻涕等盥洗的方法，便器的使用方法，尿布/纸尿裤/污染衣物的更换，便后清洁的方法，如厕习惯培养，婴幼儿大、小便异常的处理等。
　　8.活动组织与支持。一日生活和活动的安排，生活和活动环境的创设与利用，活动材料的配备，动作、语言、认知、情感与社会性等活动的组织与实施，游戏活动的支持与引导，婴幼儿行为观察与分析，婴幼儿需求的识别与回应等。

　　五、培训原则
　　（一）岗位胜任原则
　　培训应以托育机构保育人员岗位要求为重点，通过系统培训引导与自主学习反思相结合的方式，促进保育人员明晰岗位工作任务，具备胜任岗位职责的基本知识与能力。
　　（二）需求导向原则
　　培训应以托育机构保育人员在保育工作中的重点和难点为出发点，综合考虑岗位需求和发展需要，按需施教，优化培训内容，确保保育人员所学即所需、所学即所用、学用相长。
　　（三）多元方式原则
　　培训应通过专题讲座、网络研修、研讨交流、案例分析、返岗实践等多元方式，借助互联网等手段，推动托育机构保育人员理论学习和实践观摩相结合、线上学习与线下研修相结合，提高培训实效性。

　　六、培训考核
　　培训考核内容分为理论考试和实践技能考核两部分，各级卫生健康部门负责对培训效果进行抽查。

© 北大法宝：（[www.pkulaw.com](https://www.pkulaw.com/)) 专业提供法律信息、法学知识和法律软件领域各类解决方案。北大法宝为您提供丰富的参考资料，正式引用法规条文时请与标准文本核对。
欢迎查看所有[产品和服务](https://www.pkulaw.com/helps/2.html)。法宝快讯：[如何快速找到您需要的检索结果？](https://www.pkulaw.com/helps/70.html)[法宝](https://www.pkulaw.com/helps/69.html)V6有何新特色？

扫描二维码阅读原文

**原文链接：**<https://www.pkulaw.com/chl/88629c00e3275b0dbdfb.html>
